# Catarrhal Neuralgia

**Published:** 1889-12

**Authors:** A. G. Hobbs

**Affiliations:** Atlanta, Ga.; President of the American Rhinological Association


### CATARRHAL NEURALGIA.

By A. G. HOBBS, M. D., ATLANTA, GA.
President of the American Rhinological Association.

Read at the 7th annual meeting of the American Rhinological Association, at Chicago, Oct 11, 1889.

I have purposely not used the term catarrhal headache, or catarrhal browache, because both are too restrictive to cover the condition that I desire to discuss.

By catarrhal neuralgia I mean to include not only the headaches and browaches that are caused by the pressure from nasal hypertrophies or foreign growths, but all the reflex pains that have for their origin, pressure in the nasal cavities, whether they are located in the forehead, the temples, across the bridge of the nose, or over the cheek bones. That such reflex pains do occur, and have for their cause pressure in the nasal cavities, was known long ago, and I might quote authorities, but I consider that to do so before the Fellows of this Association would be an unnecessary waste of time. When we consider the great number of sensitive nerves, with their different sources that supply the nasal and adjacent mucous membranes, we could argue even from a theoretical standpoint, that reflex pains might be caused by any pressure upon these mucous membranes and their underlying tissues, but I am sure that I am warranted in saying that it is the *experience* of every one here that such pains not only exist, but have their origin in nasal hypertrophies or neoplasms. If these pains were only occasional, or of short duration, they might not demand so much of our attention, but in many cases the pains are more persistent than the neuralgias from any other cause, and they, many times, produce a more profound impression upon the general system, indicated by a sense of lassitude, a haggard expression, a mal-assimilation, and a decided loss of weight.

Much has been written recently on headaches and browaches, caused by uncorrected errors of refraction, and it cannot be denied that such reflex pains, often accompanied by nausea and dizziness, frequently have their origin in asthenopia and astigmatism. Such symptoms are reflex in character, and have their origin in the ciliary region of the eye, a part so abundantly supplied with peculiarly sensitive nerves.

While we should not be hasty in diagnosing the origin of such reflex symptoms, I do not consider it difficult to differentiate between the eye and the nose, as the cause, when the two do not co-exist.

Ocular headaches are more intermittent, usually following a prolonged use of the eyes; catarrhal neuralgia persists longer at each attack, and usually follows a choriza. Ocular headaches, if severe, are often accompanied by nausea and dizziness; catarrhal neuralgia rarely if ever causes the latter symptom.

When the eye is the cause the pain is situated in the front part of the head and temples; when the nose is the cause, the pain is not only in the head and temples, but it extends downward into the facial bones. Both causes may co-exist in the same case, when relief can be effected only by both correcting the error of refraction and reducing the nasal pressure. In such a case only a partial relief would be reached if either of the cases were neglected.

I have no statistics bearing upon the comparative frequency of catarrhal and ocular neuralgias other than my own note book of the last three years; in it I find 54 cases under the different headings: catarrhal neuralgia, ocular headache and catarrhal and ocular neuralgia combined. 36 cases occur under the 1st caption; 14 cases under the 2d and 4 cases under the 3d.

My recollection is that I have placed no one of these cases under its respective heading except where the patient applied particularly for relief from some of the above mentioned neuralgic symptoms. In the great majority of these cases the patient had no clear idea of the cause of his trouble, and, indeed, in many of them he had gone the rounds, and had taken all the anti-neuralgic remedies mentioned in therapeutics, or I might rather say, mentioned in the current circulars of enterprising drug houses. It is my opinion that the nose, more frequently than the eye, is the cause of reflex neuralgias.

After recognizing the etiology of a case exhibiting these reflex pains, the treatment is plain. On account of the severity of the pains, temporary relief should be given the patient by the application of cocaine sprays, or in case of complete stenosis, the cotton probe saturated with cocaine first, then the sprays. The effort should then be to reduce the pressure permanently by the means that is most rapid.

If the pressure be due to a polypus, extract it; if to a deflected septum, remove the deflection, and if it be, as is most likely the case, due to a hypertrophy, reduce it with the galvano-cautery, the snare, glacial acetic acid, or chromic acid. I find myself most frequently resorting to the latter. After the removal of the pressure, pain ceases to a great extent, if not entirely; then the more soothing treatment of vaselene sprays, in combination with cocaine, pinus canadensis, oil of terebene or oil of eucalyptus, daily or tri-weekly will complete the resolution.

Many cases that are essentially acute, and due to a recurrent attack of an old hypertrophy, may be relieved by a few well applied cocaine sprays, but, if left in this condition, the patient will be subject to a recurrent attack at the next choriza.

I will amplify only a few cases from my note book.

Mr. H., æt. about 30 years, commercial traveler, came to me, with excruciating pains in his forehead, temples, across the bridge of his nose and over the cheek bones of the left side; had been subject to these attacks for three years, each time he caught cold; had had no relief from medication except when insensible from morphine; attacks had lasted from three to ten days. Upon examination, the nasal passages were found closed from an acute choriza. After opening the passages, by inserting cocaine on a cotton probe, sufficiently to introduce a spray of cocaine and vaseline, relief followed almost instantly. He returned the next morning, having had but slight recurrences of pain during the interim. At this treatment, after thoroughly opening the passages with cocaine in a vaseline and eucalyptol spray, I touched the more prominent hypertrophies lightly with glacial acetic acid, and ordered the continuance of his spray at less frequent intervals. At his return the following day he reported himself entirely free from neuralgia, and when I had thoroughly removed the acid sloughs, the nasal passages were comparatively open. The patient left town that day without pursuing the treatment as I advised. Three months afterwards he felt an attack coming on in a neighboring city, when he started at once to me. After giving him relief as before, he consented to remain three weeks, during which time I treated him in the conventional way, for nasal hypertrophy. In writing to me two years afterwards, he said he had never had a recurrence of his attacks. I find in my note book thirteen other cases similar in most respects to to this one; some of them evinced some mild asthmatic symptoms, in addition to the facial and brow pains. The same trestment was effectual in all of these cases, without an exception.

I will now describe a case more chronic in its nature:

Mr. W., æt. 32 years, gave me his history as follows:

About six months previous to his first visit to me, he began having severe attacks of neuralgia in his face and forehead, when shortly afterward the pains became almost constant, and he had asthmatic symptoms at night; he was oppressed by a feeling of malaise, lost his appetite and strength, and his weight decreased from 210 to 130 pounds in a few months. After exhausting the skill of his family physician he went for six weeks to a hydropathic institution, thence to another sanitarium, for two months more. In the mean time he had taken many anti-neuralgic and tonic remedies. When I first saw him, the pains in the forehead, temples, across the bridge of the nose and over the left muscular bone were almost constant; he had nightly attacks of asthma in a mild form, but sufficient to greatly interfere with his rest; he had a haggard expression, was weak, and weight only 130 pounds.

Upon examining his nasal cavity, I found a septum deflection on the left side, and a large turbinated hypertrophy just opposite. In addition to this, a posterior nasal examination revealed a large polypus, which completely occluded the left chonæ; the right side was also in a hypertrophic condition. The left nasal passage had been almost constantly occluded for six or eight months. He said it had not been suspected before that his nose had had anything to do with his trouble. The polypus was removed with a Sajous post nasal snare and a large nuckle of the hypertrophy, with the anterior snare at the first sitting, when a partial opening was effected.

I do not consider it wonderful, when I say that he had no severe pains after the first treatment. He was instructed how to use cocaine in a vaseline spray, to prevent the recurrence of the pains. My treatments were continued daily and tri-weekly for four months, until he was entirely relieved of his nasal trouble. He began to regain his strength and weight at once, and he had at no time anything more than a trifling recurrence of his neuralgia.

It is now eighteen months since I completed his treatment, he weighs 200 pounds, and has had no sign of a neuralgic attack since.

I find in my notes, five cases, similar in many respects to this one, particularly in that they were cases of long standing before their etiology was recognized. The nasal features of these cases were not the same except that pressure existed in all.

I will describe a case to show that both nasal pressure and refractive error may be the combined cause of these reflex neuralgias: Mrs. R., æt. 36 years, came to me with the symptoms described above, together with nausea and dizziness after having made the slightest attempt to read or do any close work. I first thought I had a case of refractive error plain and simple, so I paralyzed the accommodation and corrected the astigmatism, which was moderate in degree. She was greatly relieved, to the extent that she had no nausea and dizziness after reading, which act she performed better than ever before. There seemed, however, to be but little if any, relief from her forehead and facial neuralgia, which continued to nag her at intervals.

After ten days I examined the nasal cavity which I had failed to do before, when I found a decided hypertrophy over the turbinated bones of both sides. Not until this was removed, after a treatment of about six weeks, was she entirely relieved of all her symptoms. While at a summer resort, during the following summer, she broke her glasses, and, in trying to do without them, she had a slight return of browache, and when she tried to read, a tendency to nausea and dizziness followed. After receiving her new glasses, there was no return of any of her symptoms. I find in my notes three other cases similar in many particulars to this one.

I know of no class of cases that apply to us for relief, in which treatment can give more prompt and satisfactory results, indeed when we make no mistake in the etiology, the result is absolutely sure, if we resort to means that are effectual in removing the pressure upon the terminal sensitive nerves that are distributed to the nasal mucous membrane.

